# Transcriptomic analysis elucidates the molecular processes associated with hydrogen peroxide-induced diapause termination in *Artemia*-encysted embryos

**DOI:** 10.1371/journal.pone.0247160

**Published:** 2021-02-19

**Authors:** Bonien Chen, Tah-Wei Chu, Kuohsun Chiu, Ming-Chang Hong, Tsung-Meng Wu, Jui-Wen Ma, Chih-Ming Liang, Wei-Kuang Wang

**Affiliations:** 1 Department and Graduate Institute of Aquaculture, National Kaohsiung University of Science and Technology, Kaohsiung, Taiwan; 2 Department of Oceanography, National Sun Yat-sen University, Kaohsiung, Taiwan; 3 Department of Aquaculture, National Pingtung University of Science and Technology, Pingtung, Taiwan; 4 Department of Environmental Engineering and Science, Feng Chia University, Taichung, Taiwan; Laboratoire de Biologie du Développement de Villefranche-sur-Mer, FRANCE

## Abstract

Treatment with hydrogen peroxide (H_2_O_2_) raises the hatching rate through the development and diapause termination of *Artemia* cysts. To comprehend the upstream genetic regulation of diapause termination activated by exterior H_2_O_2_ elements, an Illumina RNA-seq analysis was performed to recognize and assess comparative transcript amounts to explore the genetic regulation of H_2_O_2_ in starting the diapause termination of cysts in *Artemia salina*. We examined three groupings treated with no H_2_O_2_ (control), 180 μM H_2_O_2_ (low) and 1800 μM H_2_O_2_ (high). The results showed a total of 114,057 unigenes were identified, 41.22% of which were functionally annotated in at least one particular database. When compared to control group, 34 and 98 differentially expressed genes (DEGs) were upregulated in 180 μM and 1800 μM H_2_O_2_ treatments, respectively. On the other hand, 162 and 30 DEGs were downregulated in the 180 μM and 1800 μM H_2_O_2_ treatments, respectively. Cluster analysis of DEGs demonstrated significant patterns among these types of 3 groups. GO and KEGG enrichment analysis showed the DEGs involved in the regulation of blood coagulation (GO: 0030193; GO: 0050818), regulation of wound healing (GO:0061041), regulation of hemostasis (GO: 1900046), antigen processing and presentation (KO04612), the Hippo signaling pathway (KO04391), as well as the MAPK signaling pathway (KO04010). This research helped to define the diapause-related transcriptomes of *Artemia* cysts using RNA-seq technology, which might fill up a gap in the prevailing body of knowledge.

## Introduction

During *Artemia* diapause, the metabolic process of egg cells reduces [1], thereby decreasing energy consumption; furthermore, molecular chaperones are accustomed to prevent or decrease protein denaturation. Brine shrimp possess a solid, semipermeable outer shell that protects their embryos while making sure embryo contact with external circumstances. Brine shrimp contain trehalose, which exhibits a fluid retention capacity that protects embryos from lethal harm due to dehydration [2, 3]. Diapause termination of *Artemia* cysts was proven to be regulated by environmental elements. Robbins et al. [4] indicated that the hatching rate of *Artemia* cysts improved by 50% when subjected to 180 μM hydrogen peroxide (H_2_O_2_). Based on the experimental outcomes obtained by these studies, chemical methods may be employed to terminate cyst diapause. However, the mechanism of diapause termination has not been elucidated and therefore is a subject that warrants in-depth investigation.

Several studies on cyst diapause have been around in arthropods utilizing a transcriptomic approach to elucidate the gene expression involved with diapause or diapause termination. For examples, transcriptomic profiling uncovered significant changes in gene expressions related to gonad development, lipid synthesis, and molt regulation, suggestive of a coupling of the processes in diapause initiation in copepod *Calanus finmarchicus* [5–7]. Tu et al. [8] used the proteomic and transcriptomic tools to investigate the distinctions between diapause and non-diapause eggs at both protein and mRNA expression levels. These differentially expressed proteins and genes were associated with metabolism and cryoprotection.

Researches on *Artemia* species have exposed to exterior biotic or abiotic elements to ensure that they provide a basic background data of *Artemia* transcriptomes, these researches demonstrate that the power of RNA-seq to recognize molecules in *Artemia*, however these studies were focusing on the adult individuals [9, 10]. In this research, we uncovered brine shrimp to H_2_O_2_ to induce their diapause termination. Subsequently, RNA-seq was used| to investigate the transcriptional profiles of H_2_O_2_-induced diapause termination of *Artemia* cysts to characterize the physiological regulation of the diapause termination process at the transcriptomic level.

## Materials and methods

### *Artemia* cyst samples

The modified procedure of the acquisition of *Artemia salina* diapause cysts was according to the method provided by Robbins et al. [4]. The cysts were incubated in artificial seawater in a 15 cm Petri dish for 7 days. The shell residuals and hatched individuals were removed each day. After 7-days incubation, the unhatched-cysts were used and harvested for diapause. The diapausing cysts were then incubated in a stationary condition in a Petri dish (15 cm) with H_2_O_2_ at 0, 180 and 1800 μM for 5 h.

### RNA quantification and qualification

RNA qualification and quantification were determined by electrophoresis analysis at 1% agarose gels to monitor whether its degradation and contamination. RNA purity was measured by utilizing NanoPhotometer® spectrophotometer (IMPLEN, CA, USA). RNA concentration was determined using a Qubit® RNA Assay Kit in Qubit® 2.0 Fluorometer (Life Technologies, CA, USA). RNA integrity was assessed using the RNA Nano 6000 Assay Kit of the Agilent Bioanalyzer 2100 system (Agilent Technologies, CA, USA).

### Library preparation for transcriptome sequencing

From the RNA sample to the ultimate data, each step, including sample testing, library collecting, and sequencing, influences the quality of the data which impacts the analysis consequences. To ensure the reliability of the data, quality control (QC) is conducted at each step in this research. The workflow is shown in S1A Fig.

A total amount of 1.5 μg RNA per sample was used for the RNA sample preparations. Sequencing libraries were produced using the NEBNext® Ultra™ RNA Library Prep Kit for Illumina® (NEB, USA) following manufacturer’s guidelines, and index codes were put into attribute sequences to each sample. Briefly, mRNA was purified from total RNA using poly-T oligo-attached magnetic beads. Fragmentation was completed using divalent cations under elevated temperature in NEBNext First Strand Synthesis Reaction Buffer (5X). First strand cDNA was synthesized using random hexamer primers and M-MuLV Reverse Transcriptase. DNA polymerase I and RNase H were applied to synthesize the Second strand cDNA synthesis subsequently. The rest of the overhangs were changed into blunt ends via exonuclease/polymerase actions. After adenylation of the 3’ ends of DNA fragments, NEBNext Adaptors with hairpin loop structures were ligated for hybridization. To obtain the specific cDNA fragments of 150~200 bp in length, the library fragments were purified with the AMPure XP system (Beckman Coulter, Beverly, USA). Then, 3 μl USER Enzyme (NEB, USA) was used in combination with size-chosen, adaptor-ligated cDNA at 37°C for 15 min accompanied by 5 min at 95°C before PCR. After that, PCR was performed with Phusion High-Fidelity DNA polymerase, Universal PCR primers and Index (X) Primer. Finally, PCR products were purified (AMPure XP system), and library quality was assessed on the Agilent Bioanalyzer 2100 system. Raw cDNA sequences have been deposited in Sequence Read Archive (SRA) at NCBI BioProject resources. The following accession numbers was PRJNA664008.

### Data quality control

Raw data (raw reads) in fastq file format were processed with in-house Perl scripts. Clean data (clean reads) were obtained by dethatching reads that contain adaptors, poly-N and low-quality reads from raw data in this step. Simultaneously, the Q20, Q30, GC content and sequence duplication level of the clean reads were calculated. All downstream analyses were based on clean data with high quality.

### Transcriptome assembly

Due to the lack of a reference genome for *Artemia* samples, clean reads have to be assembled to obtain a reference sequence for the next analysis (S1B Fig). The software Trinity [11] was applied to complete the transcriptome reconstruction. The left files (read1 files) from all libraries/samples were pooled into one left.fq file, and another right files (read 2 files) were pooled into one right.fq file. Transcriptome assembly was achieved based on the left and right fq. using Trinity transcriptome assembler with min/kmer/cov set to 2 by default, and all the parameters were at default configurations.

The workflow of Trinity was described followed: Inchworm: Constructs a k-mer dictionary from all sequenced reads (k = 25), selects the most frequent seeding k-mer in the dictionary and extends the seed in both directions to create a contig assembly. Chrysalis: Chrysalis clusters minimally overlapping Inchworm contigs into sets of connected elements and constructs comprehensive de Bruijn graphs for each element. Each element defines an assortment of Inchworm contigs that will tend to be derived from alternative splice forms or closely related gene paralogs. Butterfly: Butterfly reconstructs full-length, plausible, linear transcripts by reconciling the individual de Bruijn graphs produced by Chrysalis with the original sequence reads and paired ends. Butterfly reconstructs unique transcripts for splice isoforms and paralogous genes and resolves ambiguities stemming from sequences >k bases length or from errors that are shared among transcripts. The final assembled result named as TRINITY.fasta.

### Gene functional annotation

Gene function was annotated according to the information in following databases: Nt (NCBI non-redundant nucleotide sequences),Nr (NCBI non-redundant protein sequences), KOG/COG (Clusters of Orthologous Groups of proteins), Pfam (Protein family), Swiss-Prot (A manually annotated and reviewed protein sequence database), GO (Gene Ontology) and KO (KEGG Ortholog database).

The provided information and characteristics of the seven databases are specific and following described as: Nr (NCBI non-redundant protein sequences): the formal protein sequence database of NCBI, which include protein sequence information from such databases as GenBank, Swiss-Prot, PIR (Protein Information Resource), PRF (Protein Research Foundation), and PDB (Protein Data Bank).Pfam (Protein family) is the most comprehensive collection of protein families and domains, represented as multiple sequence alignments and profile by hidden Markov model. Pfam families are classified into two categories, Pfam-A and Pfam-B, based on the reliability of annotations. Both KOG (euKaryotic Orthologous Groups) and COG (Cluster of Orthologous Groups of proteins) are derive from NCBI’s gene orthologous relationships. COG is specific to prokaryotes, while KOG is definitely specific to eukaryotes. Based on the evolutionary relationships among species, COG/KOG defined the homologous genes from different species into different ortholog clusters. Swiss-Prot was an annotated and reviewed high-quality protein sequence database which combines computed features, experimental results, and scientific conclusions. And KEGG (Kyoto Encyclopedia of Genes and Genome) is a resource for understanding the high-level functions and utilities database of each biological system.GO (Gene Ontology) is the established standard for the functional annotation of gene products and is used to classify the three following functional attributes of gene: BP: biological process, MF: molecular function, and CC: cellular component.

### Quantification of gene expression levels

The degree of gene expression was estimated by RSEM [12]; clean data had been mapped onto the assembled transcriptome, and the read count for each gene was calculated from the mapping consequences.

### Differential expression analysis

The value of read count obtained from the gene expression analysis was used for the initial data to carry out further differential expression analysis. Group differential expression analysis was conducted by using the DESeq R package (1.10.1) [13]. DESeq provides statistical routines, in which the one of the models based on the negative binomial distribution was used to determining the differential expression with digital gene expression in this study. For controlling the false discovery rate obtained from digital gene expression analysis, the P value of DESeq analysis was then adjusted by using Benjamini and Hochberg’s approach. Genes with an adjusted p-value| <0.05 was considered as differentially expression.

### Clustering and sequencing (Novogene Experimental Department)

Corset [14] functions by clustering contigs based on separates contigs and shared reads when different expression patterns observed between samples. Corset also filter out contigs with a low number of mapped reads with the information from reads (less than 10 reads). The clustering of the index-coded samples was performed on a cBot Cluster Generation System using the Illumina HiSeq PE Cluster Kit (cBot-HS) based on the manufacturer’s instructions. After cluster generation, the library preparations were sequenced on an Illumina HiSeq platform, and generated the paired-end reads.

### Gene enrichment analysis

GO enrichment analysis of the DEGs was applied on the GOseq R package-based Wallenius noncentral hypergeometric distribution [15], which can adjust the bias of gene length in DEGs.

KEGG [16] is a database for understanding the high-level functions and utilities of genes in biological systems from molecular-level information. These genes could be collected from cells, organisms and ecosystems, especially large-scale molecular datasets generated by genome sequencing and high-throughput experimental technologies (http://www.genome.jp/kegg/) could provide more reliable evidence. Software KOBAS [17] was used to evaluated the statistical enrichment of differentially expressed genes in KEGG pathways. The software and parameters used in this study for data analysis are listed in S1 Table.

## Results and discussion

### Increase of hatching rates after H_2_O_2_ treatments

After 7-d incubation of *Artemia* cysts, the un-hatched ones were collected and cultured 12 hr for identification of H_2_O_2_ treatments on the hating rates. The results showed that the hatching rate was 3.1 ± 0.90% in control group (0 μM H_2_O_2_); 47.5 ± 5.31% in low-H_2_O_2_ group (180 μM), and 4.4 ± 1.30% in high-H_2_O_2_ group (1800 μM). Robbins et al. [4] and Hong et al. [9] have demonstrated that H_2_O_2_ increases the hatching rates of *Artemia* cysts and the hatching rates decreased in higher H_2_O_2_ than in lower H_2_O_2_ treatments. The pattern of H_2_O_2_ treatments on hatching rates is similar to our study.

### Gene expression analysis

Transcriptomes of diapause cysts with or without H_2_O_2_-treated were sequenced individually, in which 26,252,846–49,563,734 raw reads were generated. The reads of adaptors and low quality were then removed by filtering process, so that there could have a clean reads for downstream analyses. The filtering process includes several steps: (1) to eliminate the reads with adaptor contamination. (2) to exclude the reads when uncertain nucleotides composition more than 10% of either reads (N > 10%). (3) to remove reads when low-quality nucleotides (base quality less than 20) constitute more than 50% of the total read. After filtering, 25,645,468–48,301,960 clean reads were obtained. The results of Kruskal-Wallis test revealed that the amounts of clean reads among the cysts with or without treating H_2_O_2_ weren’t considerably different (p = 0.2881). Therefore, the regulation of H_2_O_2_-induced diapause termination of *Artemia* cysts was related to the gene expression level rather than the amount of expressed genes. The de novo transcriptome filtered by Corset was used as a reference alignment. RSEM could map reads again to the transcriptome and quantify the expression level. S2 Table showed the mapping results. Both FPKM and RPKM used for gene expression level normalization. FPKM considered the fragment difference between these two reads of paired end reads, while RPKM considers reads: rather than using read counts, it approximates the relative abundance of transcripts regards to fragments observed from an RNA-seq experiment. That might not be represented by a single read, such as in paired-end RNA-seq experiments. In bioinformatic analysis without genome references, such as *Artemia*, the gene with FPKM>0.3 is considered to being expressed. This threshold is recommended and accepted by several prestigious journal and had been considered to be closely represent the high-level gene expression.

Clean reads were assembled by de novo method via Trinity to obtain the assembly transcriptome. To remove redundancies, the hierarchical clustering was carried out to the corset. Afterwards, the longest transcripts of each cluster were selected for unigenes. There were 114,153 transcripts and 114,057 unigenes were identified in the cyst samples (Table 1). With the priorities of genomic or transcriptomic techniques, recent researches had emphasized in-depth studies at the transcriptome level in *Artemia* organisms [9, 10, 18–20]. It was supposed that the genes involved with development or diapause termination might be correlated with the systematic changes| in each cell. Therefore, it could be the proof that there were more expressed genes in reactivated *Artemia* embryos in this study. For completeness of the *Artemia* transcriptome, the results provide valuable and supplementary information which could complete the presented library nowadays.

**Table 1:** Summary of RNA-seq metrics from *Aremia* cysts transcriptomes.

Metric	w/o H_2_O_2_	w/180μM H_2_O_2_	w/1800μM H_2_O_2_
Raw Reads	41,470,557.33 ± 9,789,097.84	36,445,362.00 ± 11,441,445.00	28,630,529.33 ± 2,565,135.63
Clean Reads	40,518,019.33 ± 9,572,949.25	35,568,416.00 ± 11,117,664.18	27,965,944.67 ± 2,407,193.29
Clean Bases	6.07 ± 1.45	5.33 ± 1.63	4.20 ± 0.40
Error (%)	0.03 ± 0.00	0.03 ± 0.00	0.03 ± 0.00
Q20	97.71 ± 0.21	97.70 ± 0.23	97.63 ± 0.12
Q30	93.37 ± 0.47	93.32 ± 0.55	93.18 ± 0.32
GC (%)	40.66 ± 0.15	40.44 ± 0.03	40.28 ± 0.19
Total transcripts	114253		
Total unigenes	114057		

Data: mean ± sd

(1) Sample name: the names of samples

(2) Raw Reads: the original sequencing reads counts

(3) Clean Reads: number of reads after filtering

(4) Clean Bases: clean reads number multiply read length, saved in G unit

(5) Error Rate: average sequencing error rate, which is calculated by Qphred = -10log10(e)

(6) Q20: percentages of bases whose correct base recognition rates are greater than 99% in total bases

(7) Q30: percentages of bases whose correct base recognition rates are greater than 99.9% in total bases

(8) GC content: percentages of G and C in total bases

### Gene functional annotation

**Fig 1:**
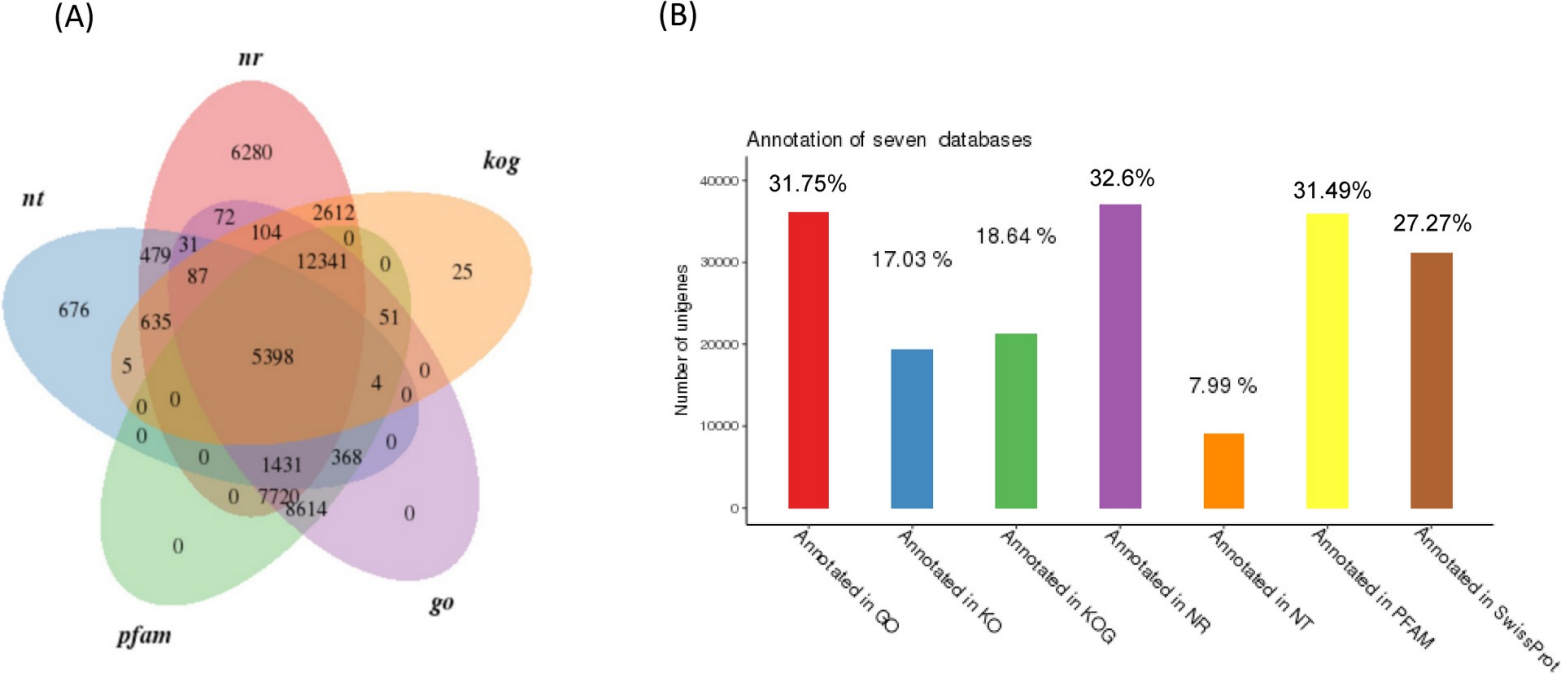
The number and percentage of identified unigenes based on the selected databases. (A) the venn diagram is mapped with 5 selected database annotation result. (B) the number and percentage of identified unigenes through 7 databases.

**Fig 2:**
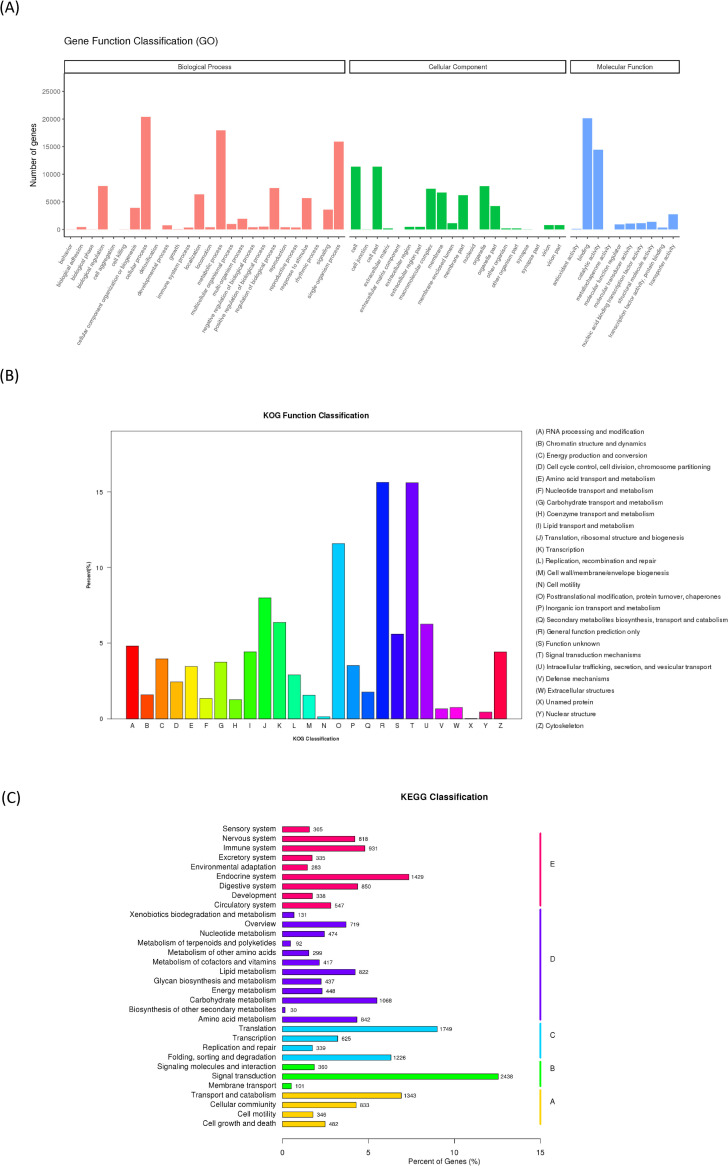
Functional annotation results of identified unigenes. (A) Gene Ontology annotation that successfully annotated genes will group into three main GO domains: biological process (BP), cellular component (CC), molecular function (MF). (B) Karyotic Orthologous Groups (KOG) classification of putative proteins (C) Kyoto Encyclopedia of Genes and Genome (KEGG) pathway contains: metabolism, genetic information processing, environmental information processing, cellular processes, organismal systems and human diseases. Human diseases results were removed in the result of KOG and KEGG pathways.

To achieve comprehensive gene functional annotation, seven databases were applied by Novogene. These databases were utilized for functional annotation of identified unigenes, and the statistics of successfully annotated genes in this research by the database are shown in Fig 1 and S3 Table. Of the 114,057 unigenes, the number of genes successfully annotated in at least one database against NT, NR, KO, Swiss Prot, PFAM, KOG and GO was 47,020 (41.22%). The summary of gene annotations based on each database is listed in S3 Table. The functional annotation of these genes according to KOG, KEGG and GO classifications is demonstrated in Fig 2. In GO, the most top five annotated genes were linked to 1) cellular process, 2) binding, 3) metabolic process, 4) single-organism process, and 5) catalytic activity. Those in KOG were related linked to 1) general function mechanism only, 2) signal transduction mechanisms, 3) posttranslational modification, protein turnover, chaperones, 4) translation, and 5) ribosomal structure and biogenesis. In the KEGG pathway, these genes were involved with 1) signal transduction, 2) translation, 3) the endocrine system, 4) transport and catabolism, and 5) proteins folding, sorting and degradation.

The patterns of GO, KOG and KEGG, classifications were consistent with those of the *A*. *sinica* transcriptome [10], except for the amount of expressed genes. Thus, the additional genes recognized from this study provided a supplementary draft for *Artemia* transcriptomes.

### Differential gene expression related to H_2_O_2_-induced diapause termination

**Fig 3:**
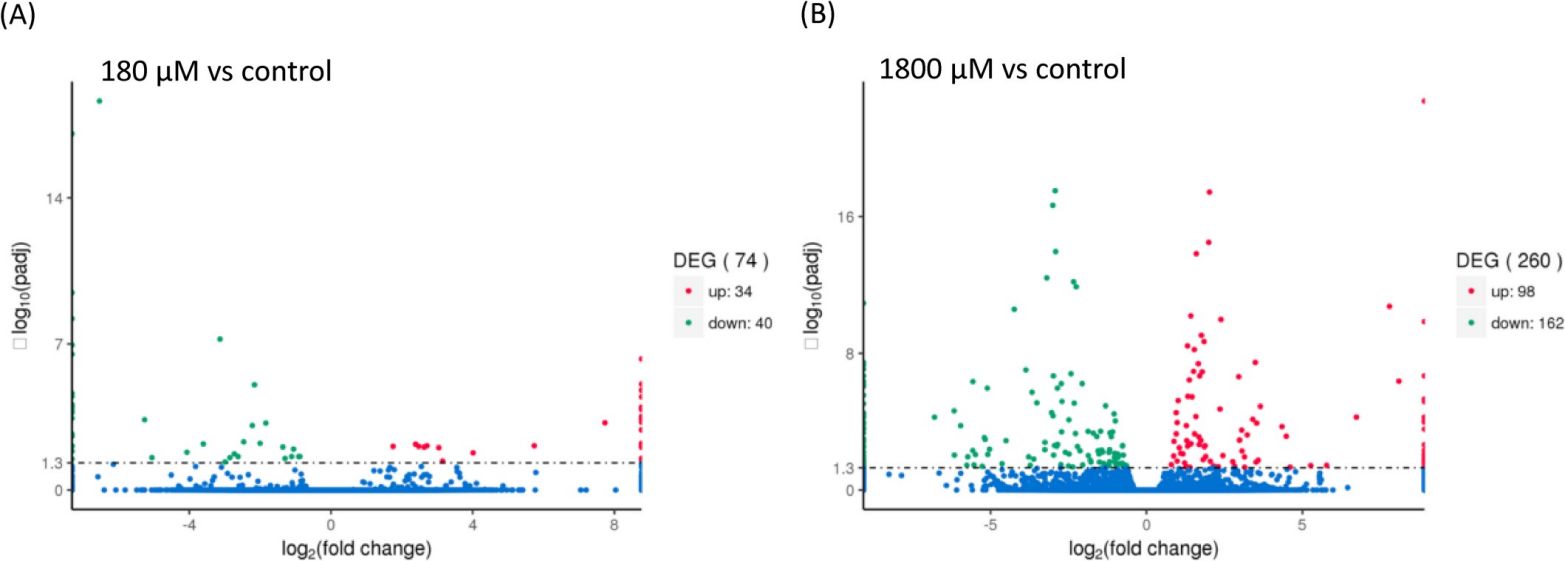
Volcano plots of differentially expressed genes associated with H_2_O_2_ treatments. The X axis represents fold change, while the Y axis indicates the significance of differential expression. (A) 180 μM vs control. (B) 1800 μM vs control.

Volcano plots were used to infer the overall distribution of differentially expressed genes (DEGs), and the number of DEGs for each paired comparison among the control (w/o H_2_O_2_) and H_2_O_2_ treatments (w/180- and 1,800-μM H_2_O_2_) are shown in Fig 3. When compared to control group, 34 and 98 DEGs were upregulated in the 180- and 1800-μM H_2_O_2_-treated groups, respectively. Furthermore, 40 and 162 DEGs were downregulated in the 180- and 1800-μM H_2_O_2_-treated groups, respectively. DEGs referring to H_2_O_2_-induced up- or downregulation compared to the control group were listed in Table 2.

**Table 2:** Up- and down-regulated genes under H_2_O_2_ treatments in *Artemia* diapause cysts.

Gene Name	180μM	1800μM	UniGenes	Term
VARS, valS FARSB	↑		Cluster2579.61530	Aminoacyl-tRNA biosynthesis
CYP15A1_C1	↑		Cluster2579.21243	Insect hormone biosynthesis
MPDZ, MUPP1, Patj	↑		Cluster2579.55288	Tight junction
ATG12	↑		Cluster2579.41553	Regulation of autophagy/RIG-I-like receptor signaling pathway/FoxO signaling pathway
E2F4_5	↑		Cluster2579.24946	TGF-beta signaling pathway/Cell cycle
CTTN, EMS1	↑		Cluster2579.53583	Shigellosis/Pathogenic *Escherichia coli* infection/Bacterial invasion of epithelial cells/Proteoglycans in cancer
GYS	↑	↑	Cluster2579.13656	Starch and sucrose metabolism/AMPK signaling pathway/Insulin signaling pathway/PI3K-Akt signaling pathway
MLL1	↑		Cluster2579.7161	Lysine degradation/Transcriptional misregulation in cancer
E1.14.11.2	↑		Cluster2579.60582	Arginine and proline metabolism
MPDZ, MUPP1, Patj	↑		Cluster2579.55288	Hippo signaling pathway—fly
CACNA1H	↑		Cluster2579.6210	Circadian entrainment/Calcium signaling pathway/Calcium signaling pathway/MAPK signaling pathway
RASAL2	↑	↑	Cluster2579.27404	Ras signaling pathway
K16330	↑		Cluster2579.24942	Pyrimidine metabolism
CTSO	↑		Cluster2579.28731	Lysosome
NDUFS3	↑		Cluster2579.45089	Non-alcoholic fatty liver disease (NAFLD)/Oxidative phosphorylation/Parkinson’s disease/Alzheimer’s disease/Huntington’s disease
E3.5.4.3, guaD	↓		Cluster2579.11205	Purine metabolism
PDE1	↓		Cluster2579.6962	Morphine addiction/Calcium signaling pathway/Purine metabolism
PDE4	↓		Cluster2579.18105	cAMP signaling pathway
BMPR2	↓		Cluster2579.35766	Cytokinecytokine receptor interaction/TGF-beta signaling pathway/Signaling pathways regulating pluripotency of stem cells/MicroRNAs in cancer/Hippo signaling pathway
TBK1	↓		Cluster2579.19502	RIG-I-like receptor signaling pathway/Cytosolic DNA-sensing pathway/Toll-like receptor signaling pathway/Hepatitis C/Measles/Hepatitis B/Ras signaling pathway/Herpes simplex infection/Influenza A/Epstein-Barr virus infection
CACNA2DN CACNA1N	↓		Cluster2579.54638	MAPK signaling pathway
			Cluster2579.67327	
E3.2.1.24	↓		Cluster2579.64238	Other glycan degradation
htpG, HSP90A	↓		Cluster25290.0	NOD-like receptor signaling pathway/Plantpathogen interaction/Prostate cancer/Antigen processing and presentation/Progesteronemediated oocyte maturation/Estrogen signaling pathway/PI3K-Akt signaling pathway/Pathways in cancer/Protein processing in endoplasmic reticulum
GIT1	↓		Cluster2579.39583	Epithelial cell signaling in *Helicobacter pylori* infection/Regulation of actin cytoskeleton
SMC1	↓		Cluster2579.34889	Cell cycle yeast/Cell cycle
EIF6	↓	↓	Cluster2579.23978	Ribosome biogenesis in eukaryotes
LSM4	↓		Cluster2579.41833	RNA degradation/Spliceosome
CYP18A1		↑	Cluster2579.33894	Insect hormone biosynthesis
SCD, desC GYS		↑	Cluster30892.0	AMPK signaling pathway
			Cluster2579.13656	
NR5A2, FTF		↑	Cluster2579.27421	Maturity onset diabetes of the young
A2M		↑	Cluster2579.38707	Complement and coagulation cascades
BRCA2, FANCD1		↑	Cluster2579.56302	Homologous recombination/Fanconi anemia pathway/Pancreatic cancer/Pathways in cancer
EXTL3		↑	Cluster2579.55901	Glycosaminoglycan biosynthesis heparan sulfate/heparin
SCD, desC		↑	Cluster30892.0	Biosynthesis of unsaturated fatty acids/PPAR signaling pathway/Fatty acid metabolism
deoC, DERA		↑	Cluster2579.69015	Pentose phosphate pathway
SNW1, SKIIP, SKIP		↑	Cluster2579.36513	Notch signaling pathway/Viral carcinogenesis/Spliceosome/Epstein-Barr virus infection
ELOVL7		↑	Cluster2579.48704	Fatty acid elongation
NOS1		↑	Cluster2579.34982	Amyotrophic lateral sclerosis (ALS)/Long-term depression/Arginine and proline metabolism/Circadian entrainment/Salivary secretion/Phagosome/Alzheimer’s disease
GPD1		↑	Cluster2579.39411	Glycerophospholipid metabolism
nagA, AMDHD2		↑	Cluster2579.16917	Amino sugar and nucleotide sugar metabolism
FARSB, pheT		↑	Cluster2579.35161	Aminoacyl-tRNA biosynthesis
EYA1		↑	Cluster2579.43795	Transcriptional misregulation in cancer
HSPA HSPA1_8		↓	Cluster19454.1	Antigen processing and presentation/Estrogen signaling pathway
			Cluster2579.2811	
			Cluster2579.68593	
			Cluster15619.0	
htpG, HSP90, AhtpG		↓	Cluster25290.0	NOD-like receptor signaling pathway/Plant-pathogen interaction/Prostate cancer/Progesteronemediated oocyte maturation/Pathways in cancer
			Cluster24430.0	
EEF1A		↓	Cluster28693.0	Legionellosis/Toxoplasmosis/Measles/Spliceosome/Endocytosis/Influenza A
			Cluster30677.0	
CACNB2,CACNA2DN MKNK, MNK MAP3K13, LZK		↓	Cluster2579.5710	MAPK signaling pathway
			Cluster2579.54638	
			Cluster2579.41607	
			Cluster2579.34400	
LIMD1,ZDHHC9_14_1 MPDZ, MUPP1 Patj AJUBA, WTIP		↓	Cluster2579.46774	Hippo signaling pathway—fly
			Cluster2579.55157	
			Cluster2579.20389	
HSPA5, PDIA6, TXNDC		↓	Cluster2579.13853	Protein processing in endoplasmic reticulum
			Cluster2579.21006	
			Cluster35149.0	
DIAPH1 DOCK		↓	Cluster2579.53371	Shigellosis
			Cluster2579.57004	
STIP1		↓	Cluster2579.68691	Prion diseases/Protein export/Thyroid hormone synthesis
CACNB2		↓	Cluster2579.5710	Arrhythmogenic right ventricular cardiomyopathy (ARVC)/Hypertrophic cardiomyopathy (HCM)/Dilated cardiomyopathy
TFIIE1, GTF2E1, TFA1 tfe			Cluster15619.0	Epstein-Barr virus infection
			Cluster2579.25410	
glnA, GLUL E4.1.3.1, ace		↓	Cluster28996.0	Glyoxylate and dicarboxylate metabolism
			Cluster2579.67175	
			Cluster14244.0	
VAV, DIAPH1		↓	Cluster2579.54232	Focal adhesion, Leukocyte transendothelial migration
			Cluster2579.53371	
clpX, CLPX		↓	Cluster2579.41888	Cell cycle Caulobacter
PPP2R3, pckA, PEPCK		↓	Cluster2579.46613	PI3K-Akt signaling pathway/mRNA surveillance pathway/Dopaminergic synapse/AMPK signaling pathway
			Cluster2579.37834	
DOCK1		↓	Cluster2579.57004	Bacterial invasion of epithelial cells
CACNB2 RGS2		↓	Cluster2579.5710	Oxytocin signaling pathway
			Cluster2579.8158	
MPDZ, MUPP1 Patj		↓	Cluster2579.55157	Tight junctio
EYA1		↓	Cluster2579.41820	Transcriptional misregulation in cancer
SIN3A		↓	Cluster2579.35774	Thyroid hormone signaling pathway/Viral myocarditis/Pathogenic Escherichia coli infection/Salmonella infection/Adherens junction/Phototransduction fly/Vibrio cholerae infection/Platelet activation
AJUBA, LIMD1 WTIP		↓	Cluster2579.20389	Hippo signaling pathway
TOP3		↓	Cluster2579.31547	Homologous recombination/Fanconi anemia pathway
SETDB1, OTX1		↓	Cluster2579.30770	Signaling pathways regulating pluripotency of stem cells
			Cluster2579.52125	
MKNK, MNK		↓	Cluster2579.41607	HIF-1 signaling pathway
glnA, GLUL		↓	Cluster2579.67175	Nitrogen metabolism/Arginine and proline metabolism/Glutamatergic synapse/Biosynthesis of amino acids/Two-component system
Cl		↓	Cluster2579.62889	Hedgehog signaling pathway
dnaK, DCP1B		↓	Cluster2579.61706	RNA degradation
			Cluster2579.42146	
VAV		↓	Cluster2579.54232	B cell receptor signaling pathway/Fc epsilon RI signaling pathway/Natural killer cell mediated cytotoxicity/Chemokine signaling pathway/cAMP signaling pathway
mutY		↓	Cluster2579.58355	Base excision repair
EEF1A, XPOT, EEF1A		↓	Cluster28693.0	RNA transport
			Cluster2579.30436 Cluster30677.0	
PPT		↓	Cluster2579.1265	Fatty acid elongation/Fatty acid metabolism
PTGDS		↓	Cluster21276.0	Arachidonic acid metabolism
DNM1L		↓	Cluster2579.40879	TNF signaling pathway
PPP2R3		↓	Cluster2579.46613	Adrenergic signaling in cardiomyocytes
RP-S6e, RPS6		↓	Cluster14365.0	Proteoglycans in cancer, Ribosome
ABCA3		↓	Cluster2579.40956	ABC transporters
UBE1, UBA1, ATPeF1B, ATP5B, ATP2, SIN3A,		↓	Cluster2579.42277	Parkinson’s disease, Huntington’s disease
			Cluster31495.0	
KCNMA1		↓	Cluster2579.9471	Insulin secretion/Phosphatidylinositol signaling system/Vascular smooth muscle contraction/Pancreatic secretion
KIDINS220, ARMS		↓	Cluster2579.42798	Neurotrophin signaling pathway
dnaK		↓	Cluster2579.61706	Tuberculosis
CACYBP, SIP		↓	Cluster16736.0	Wnt signaling pathway
TFIIE1, GTF2E1, TFA1		↓	Cluster2579.25410	Viral carcinogenesis
ATPeF1B, ATP5B, ATP2		↓	Cluster31495.0	Oxidative phosphorylation/Alzheimer’s disease

Cluster analysis was used to discover genes with similar expression patterns under our experimental conditions (S2 Fig). By clustering genes with comparable expression patterns, it may be possible to discern unknown functions of previously characterized genes or the features of unknown genes. In hierarchical clustering, areas of different colors denote different groups (clusters) of genes, and each group may have similar functions or take part in the same biological process. It showed different patterns among these three groups significantly. GO and KEGG enrichment analyses were further utilized to gather genes into biological functions or pathways through bioinformatic databases and statistical tools. The GO enrichment analysis helped to elucidate the gene function of differentially expressed genes, while the KEGG enrichment analysis also helped to elucidate the metabolic pathways where the genes were involved.

Compared to the control group, the annotations of up- and downregulated DEGs in H_2_O_2_ treatments by GO and KEGG enrichment analysis are listed in Tables 3 and 4.

**Table 3:** Significantly Enriched GO terms in DEGs.

GO accession	Description	Term type	Over represented p-Value	DEG item	DEG list
GO:0030193	regulation of blood coagulation	biological process	0.0010239	2	62
GO:0050818	regulation of coagulation	biological process	0.0010239	2	62
GO:0061041	regulation of wound healing	biological process	0.0010239	2	62
GO:1900046	regulation of hemostasis	biological process	0.0010239	2	62

(1) Gene Ontology entry.

(2) Detailed description of Gene Ontology.

(3) GO types, including cellular component, biological process and molecular function.

(4) p-value in hypergenometric test.

(5) Number of DEGs with GO annotation.

(6) Number of all reference genes with GO annotation.

**Table 4:** KEGG Enrichment of DEGs.

Term	Pathway ID	Sample number	Background number
Antigen processing and presentation	ko04612	6	148
Hippo signaling pathway	ko04391	5	196
MAPK signaling pathway	ko04010	7	366

(1) Term: Description of KEGG pathways

(2) Pathway ID: KEGG ID.

(3) Sample Number: Number of DGEs with pathway anntation.

(4) Background Number: Number of all reference genes with pathway annotation.

**Fig 4:**
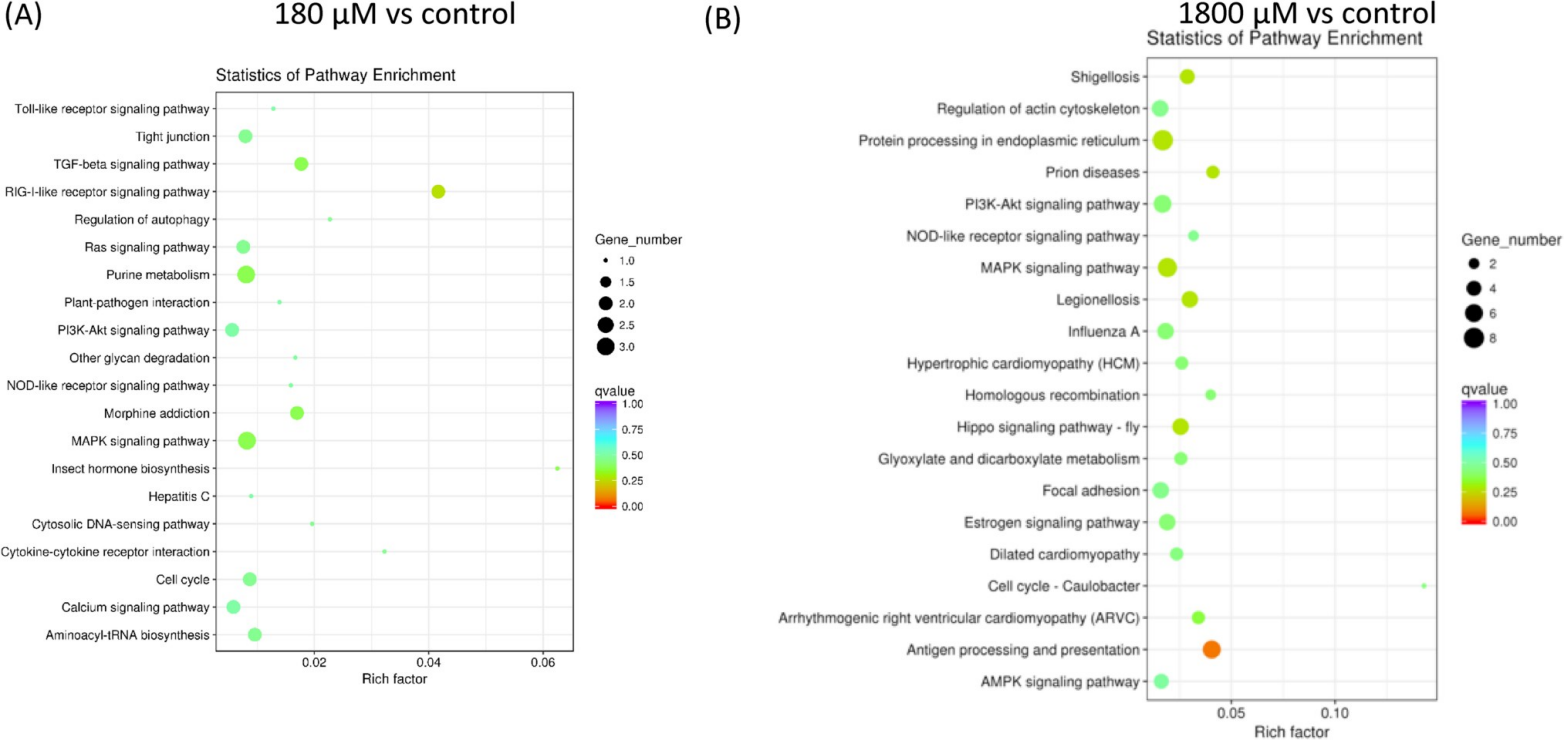
Scatter diagram of top 20 pathway enrichments for DEGs. The X axis corresponds to the Rich factor that is the ratio of DEGs to all the genes in this pathway. The Y axis represents the different pathway. (A) 180 μM vs control. (B) 1800 μM vs control.

GO enrichment analysis of DEGs showed that the biological functions of DEGs were involved in the regulation of blood coagulation, coagulation, wound healing and hemostasis (Table 3). The interactions of multiple genes could be involved in certain biological functions. As KEGG is a collection of manually curated databases regarding genomes, biological pathways, diseases, drugs, and chemical substances, KEGG was a powerful tool in utilized for pathway enrichment analysis that identified significantly enriched metabolic pathways or signal transduction pathways associated with differentially expressed genes compared with the control group (Table 4 and Fig 4). The degree of KEGG enrichment was measured by the Rich factor that refers to the ratio of the amount of DEGs in the pathway and the amount of all genes annotated in the pathway. The Q-value is the p-value after normalization, and its range is [0,1]. The smaller the q-value is definitely representing the significance of the enrichment analysis. The top 20 significantly upregulated DEGs enriched pathways in H_2_O_2_ treatments compared with the control group are shown in Fig 4.

In previous studies, most of them on genes related to the regulation of diapause termination in Artemia cysts focused on genes related to adversity resistance [9, 21–24]. For examples, these reported genes or proteins are involved in the regulation of diapause termination have been elucidated including HSP70 [25], HSP p26 [26, 27], ArHSPs [28–32] and heat shock factor 1 (Hsf1) [33, 34]. In addition, There are also many recent studies on late embryogenesis abundant (LEA) proteins in their roles on *Artemia* [35–42]. The late embryogenesis abundant (LEA) proteins in plants, bacteria and invertebrates can prevent organisms from protein aggregation caused by low temperature-related dryness or osmotic pressure, and then LEA proteins prevent damage caused by drying, cold or high salinity of a variety of organisms. Thus, diapause termination and adversity resistance have many related mechanisms in regulation.

These previous studies provide us with a protective mechanism against adversity to explore the regulation of diapause termination. NGS was used to study the gene expressions of *Artemia* under high salinity, and it was found that high salinity would increase the influence of cell cycle arrest through La-related protein and many genes related to lipid transporters [9]. The DEGs involved in binding and catalytic activity were observed to be upregulated during initial development in *Artemia* cysts [43]. This finding is similar to the results obtained in this research in reactivated diapaused cysts, in which the physiological process for development or diapause termination is close to binding and catalysis. In our previous study, with a metabolomic approach to elucidate the metabolic regulation of H_2_O_2_-induced diapause termination. MetaboAnalyst pathway analysis showed the pathway impact of metabolites in groups displaying amino acid degradation and biosynthesis and aminoacyl-tRNA biosynthesis [44]. Among them, aminoacyl-tRNA biosynthesis that was also identified and up-regulated in H_2_O_2_ treatments in this study at transcriptional level. Additionally, we also discovered the other regulation pathways involved in H_2_O_2_ treatments which were the first findings in diapause termination of *Artmeia* cysts.

In this study, we analyzed global gene expressions in *Artemia* cysts in the use of H2O2 to induce diapause termination. These findings were different from the previous studies that focused on genes or proteins involved in the regulation or responses against environmental stress. We found that these DEGs are involved in coagulation regulation and wounds. healing, hemostasis, antigen processing and presentation, Hippo signaling pathway and MAPK signaling pathway. Therefore, this work shows that diapause termination of *Artemia* cysts is regulated by other pathways which are important on known or novel mechanisms in diapause termination.

## Conclusions

In previous studies, most of the diapause termination focused on the regulation related to anti-stress responses, however, in this study, apply with a RNA-seq approach to discover upstream genetic regulation mechanisms that have not been described in *Artemia* cysts. The novel findings show a lot of genes and pathways which provide a new view to diapause termination of *Artemia* cysts.

## Supporting information

S1 Fig The analysis workflow of transcriptome assembly without a reference genome. (TIF)

S2 Fig Heat map for cluster analysis of differentially expressed genes. (TIF)

S1 Table Software and parameters used in this study. (DOCX)

S2 Table Overview of the alignment situation. (DOCX)

S3 Table The database for gene annotation and the annotated percentage. (DOCX)

## References

[1] Clegg J. Embryos of *Artemia franciscana* survive four years of continuous anoxia: the case for complete metabolic rate depression. J Exp Biol. 1997;200(3):467–75. 9318130 10.1242/jeb.200.3.467

[2] Clegg JS, Jackson SA. Aerobic heat shock activates trehalose synthesis in embryos of *Artemia franciscana*. FEBS letters. 1992;303(1):45–7. 10.1016/0014-5793(92)80474-u 1592115

[3] Elbein AD, Pan Y, Pastuszak I, Carroll D. New insights on trehalose: a multifunctional molecule. Glycobiology. 2003;13(4):17R–27R. 10.1093/glycob/cwg047 12626396

[4] Robbins HM, Van Stappen G, Sorgeloos P, Sung YY, MacRae TH, Bossier P. Diapause termination and development of encysted *Artemia* embryos: roles for nitric oxide and hydrogen peroxide. J Exp Biol. 2010;213(Pt 9):1464–70. 10.1242/jeb.041772 20400630

[5] Tarrant AM, Baumgartner MF, Hansen BH, Altin D, Nordtug T, Olsen AJ. Transcriptional profiling of reproductive development, lipid storage and molting throughout the last juvenile stage of the marine copepod Calanus finmarchicus. Front Zool. 2014;11(1):91 10.1186/s12983-014-0091-8 25568661 PMC4285635

[6] Tarrant AM, Baumgartner MF, Lysiak NS, Altin D, Storseth TR, Hansen BH. Transcriptional profiling of metabolic transitions during development and diapause preparation in the copepod *Calanus finmarchicus*. Integr Comp Biol. 2016;56(6):1157–69. 10.1093/icb/icw060 27252191

[7] Tarrant AM, Baumgartner MF, Lysiak NSJ, Hansen BH, Altin D, Nordtug T, et al Transcriptomics of diapause and lipid accumulation in the marine copepod Calanus finmarchicus. Integr Comp Biol. 2016;56:E218-E. 10.1093/icb/icw060 27252191

[8] Tu X, Wang J, Hao K, Whitman DW, Fan Y, Cao G, et al Transcriptomic and proteomic analysis of pre-diapause and non-diapause eggs of migratory locust, Locusta migratoria L. (Orthoptera: Acridoidea). Sci Rep. 2015;5:11402 10.1038/srep11402 26091374 PMC4650673

[9] De Vos S, Van Stappen G, Sorgeloos P, Vuylsteke M, Rombauts S, Bossier P. Identification of salt stress response genes using the *Artemia* transcriptome. Aquaculture. 2019;500:305–14.

[10] Zhang YL, Wang D, Zhang Z, Wang ZP, Zhang DC, Yin H. Transcriptome analysis of Artemia sinica in response to Micrococcus lysodeikticus infection. Fish Shellfish Immun. 2018;81:92–8. 10.1016/j.fsi.2018.06.033 30006042

[11] Grabherr MG, Haas BJ, Yassour M, Levin JZ, Thompson DA, Amit I, et al Full-length transcriptome assembly from RNA-Seq data without a reference genome. Nature Biotechnology. 2011;29(7):644–52. 10.1038/nbt.1883 21572440 PMC3571712

[12] Li B, Dewey CN. RSEM: accurate transcript quantification from RNA-Seq data with or without a reference genome. BMC Bioinformatics. 2011;12(1):323. 21816040 10.1186/1471-2105-12-323 PMC3163565

[13] Anders S, Huber W. W. Differential expression analysis for sequence count data. Genome Biol 11, R106 2010 10.1186/gb-2010-11-10-r106 20979621 PMC3218662

[14] Davidson NM, Oshlack A. Corset: enabling differential gene expression analysis for de novoassembled transcriptomes. Genome Biology. 2014;15(7):410 10.1186/s13059-014-0410-6 25063469 PMC4165373

[15] Young MD, Wakefield MJ, Smyth GK, Oshlack A. Gene ontology analysis for RNA-seq: accounting for selection bias. Genome Biology. 2010;11(2):R14 10.1186/gb-2010-11-2-r14 20132535 PMC2872874

[16] Kanehisa M, Araki M, Goto S, Hattori M, Hirakawa M, Itoh M, et al KEGG for linking genomes to life and the environment. Nucleic Acids Res. 2008;36(Database issue):D480–D4. 10.1093/nar/gkm882 18077471 PMC2238879

[17] Mao X, Cai T, Olyarchuk JG, Wei L. Automated genome annotation and pathway identification using the KEGG Orthology (KO) as a controlled vocabulary. Bioinformatics. 2005;21(19):3787–93. 10.1093/bioinformatics/bti430 15817693

[18] Han X, Xu R, Zheng Y, Gao M, Sui L. Development of EST-SSR markers and genetic diversity analysis among three *Artemia* species from different geographic populations. Crustaceana. 2019;92:841–51.

[19] Valenzuela-Miranda D, Gallardo-Escarate C, Valenzuela-Munoz V, Farlora R, Gajardo G. Sex-dependent transcriptome analysis and single nucleotide polymorphism (SNP) discovery in the brine shrimp Artemia franciscana. Mar Genom. 2014;18:151–4. 10.1016/j.margen.2014.10.007 25450167

[20] Yi XL, Zhang KK, Liu RY, Giesy JP, Li ZC, Li WT, et al Transcriptomic responses of Artemia salina exposed to an environmentally relevant dose of Alexandrium minutum cells or Gonyautoxin2/3. Chemosphere. 2020;238 10.1016/j.chemosphere.2019.124661 31472350

[21] Clegg JS. Stress-related proteins compared in diapause and in activated, anoxic encysted embryos of the animal extremophile, *Artemia franciscana*. J Insect Physiol. 2011;57: 660–4. 10.1016/j.jinsphys.2010.11.023 21147115

[22] MacRae TH. Gene expression, metabolic regulation and stress tolerance during diapause. Cell Mol Life Sci. 2010;67:2405–24. 10.1007/s00018-010-0311-0 20213274 PMC11115916

[23] MacRae TH. Stress tolerance during diapause and quiescence of the brine shrimp, *Artemia*. Cell Stress Chaperones. 2016;21(1):9–18. 10.1007/s12192-015-0635-7 26334984 PMC4679736

[24] Zhu XJ, Feng CZ, Dai ZM, Zhang RC, Yang WJ. AMPK alpha subunit gene characterization in *Artemia* and expression during development and in response to stress. Stress. 2007;10(1):53–63. 10.1080/10253890601130773 17454967

[25] Iryani MTM, Sorgeloos P, Danish-Daniel M, Tan MP, Wong LL, Mok WJ, et al Cyst viability and stress tolerance upon heat shock protein 70 knockdown in the brine shrimp *Artemia franciscana*. Cell Stress Chaperones. 2020; 10.1007/s12192-020-01113-0 32383141 PMC7591639

[26] King AM, MacRae TH. The small heat shock protein p26 aids development of encysting Artemia embryos, prevents spontaneous diapause termination and protects against stress. Plos One. 2012;7:e43723 10.1371/journal.pone.0043723 22952748 PMC3428274

[27] Malitan HS, Cohen AM, MacRae TH. Knockdown of the small heat-shock protein p26 by RNA interference modifies the diapause proteome of *Artemia franciscana*. Biochem Cell Biol. 2019;97:471–9. 10.1139/bcb-2018-0231 30620618

[28] Jiang G, Rowarth NM, Panchakshari S, MacRae TH. ArHsp40, a type 1 J-domain protein, is developmentally regulated and stress inducible in post-diapause *Artemia franciscana*. Cell Stress Chaperones. 2016;21:1077–88. 10.1007/s12192-016-0732-2 27581971 PMC5083676

[29] Qiu Z, MacRae TH. ArHsp22, a developmentally regulated small heat shock protein produced in diapause-destined Artemia embryos, is stress inducible in adults. FEBS J. 2008;275:3556–66. 10.1111/j.1742-4658.2008.06501.x 18537825

[30] Qiu Z, Macrae TH. ArHsp21, a developmentally regulated small heat-shock protein synthesized in diapausing embryos *of Artemia franciscana*. Biochem J. 2008;411:605–11. 10.1042/BJ20071472 18095938

[31] Rowarth NM, MacRae TH. ArHsp40 and ArHsp40-2 contribute to stress tolerance and longevity in *Artemia franciscana*, but only ArHsp40 influences diapause entry. J Exp Biol. 2018;221(Pt 20). 10.1242/jeb.189001 30158133

[32] Rowarth NM, MacRae TH. Post-diapause synthesis of ArHsp40-2, a type 2 J-domain protein from Artemia franciscana, is developmentally regulated and induced by stress. Plos One. 2018;13:e0201477 10.1371/journal.pone.0201477 30048537 PMC6062144

[33] Tan J, MacRae TH. Stress tolerance in diapausing embryos of Artemia franciscana is dependent on heat shock factor 1 (Hsf1). Plos One. 2018;13:e0200153 10.1371/journal.pone.0200153 29979776 PMC6034868

[34] Tan J, MacRae TH. The synthesis of diapause-specific molecular chaperones in embryos of Artemia franciscana is determined by the quantity and location of heat shock factor 1 (Hsf1). Cell Stress Chaperones. 2019;24:385–92. 10.1007/s12192-019-00971-7 30701477 PMC6439115

[35] Boswell LC, Moore DS, Hand SC. Quantification of cellular protein expression and molecular features of group 3 LEA proteins from embryos of *Artemia franciscana*. Cell Stress Chaperones. 2014;19:329–41. 10.1007/s12192-013-0458-3 24061850 PMC3982030

[36] LeBlanc BM, Hand SC. A novel group 6 LEA protein from diapause embryos of *Artemia franciscana* is cytoplasmically localized. Tissue Cell. 2020;67:101410 10.1016/j.tice.2020.101410 32835943

[37] LeBlanc BM, Le MT, Janis B, Menze MA, Hand SC. Structural properties and cellular expression of AfrLEA6, a group 6 late embryogenesis abundant protein from embryos of *Artemia franciscana*. Cell Stress Chaperones. 2019;24:979–90. 10.1007/s12192-019-01025-8 31363993 PMC6717223

[38] Toxopeus J, Warner AH, MacRae TH. Group 1 LEA proteins contribute to the desiccation and freeze tolerance of *Artemia franciscana* embryos during diapause. Cell Stress Chaperones. 2014;19:939–48. 10.1007/s12192-014-0518-3 24846336 PMC4389855

[39] Warner AH, Chakrabortee S, Tunnacliffe A, Clegg JS. Complexity of the heat-soluble LEA proteome in *Artemia* species. Comp Biochem Physiol Part D Genomics Proteomics. 2012;7:260–7. 10.1016/j.cbd.2012.04.002 22595823

[40] Warner AH, Guo ZH, Moshi S, Hudson JW, Kozarova A. Study of model systems to test the potential function of Artemia group 1 late embryogenesis abundant (LEA) proteins. Cell Stress Chaperones. 2016;21:139–54. 10.1007/s12192-015-0647-3 26462928 PMC4679747

[41] Wu G, Zhang H, Sun J, Liu F, Ge X, Chen WH, et al Diverse LEA (late embryogenesis abundant) and LEA-like genes and their responses to hypersaline stress in post-diapause embryonic development of *Artemia franciscana*. Comp Biochem Physiol B Biochem Mol Biol. 2011;160:32–9. 10.1016/j.cbpb.2011.05.005 21620991

[42] Zhao W, Yao F, Zhang M, Jing T, Zhang S, Hou L, et al The potential roles of the G1LEA and G3LEA proteins in early embryo development and in Response to low temperature and High salinity in *Artemia sinica*. Plos One. 2016;11:e0162272 10.1371/journal.pone.0162272 27603306 PMC5014412

[43] Chen WH, Ge X, Wang W, Yu J, Hu S. A gene catalogue for post-diapause development of an anhydrobiotic arthropod *Artemia franciscana*. BMC Genomics. 2009;10:52 10.1186/1471-2164-10-52 19173719 PMC2649162

[44] Hong MC, Ding S, Lin CC, Chu TW, Chiu KH. NMR-based untargeted metabolomic study of hydrogen peroxide-induced development and diapause termination in brine shrimp. Comp Biochem Physiol Part D Genomics Proteomics. 2017;24:118–26. 10.1016/j.cbd.2017.09.001 28982093

