# Supplementary figures and images for: Transcriptomic analysis elucidates the molecular processes associated with hydrogen peroxide-induced diapause termination in Artemia-encysted embryos
Source: PLoS One. 2021 Feb 19;16(2):e0247160. doi: 10.1371/journal.pone.0247160 (PMC7894940; doi:10.1371/journal.pone.0247160)

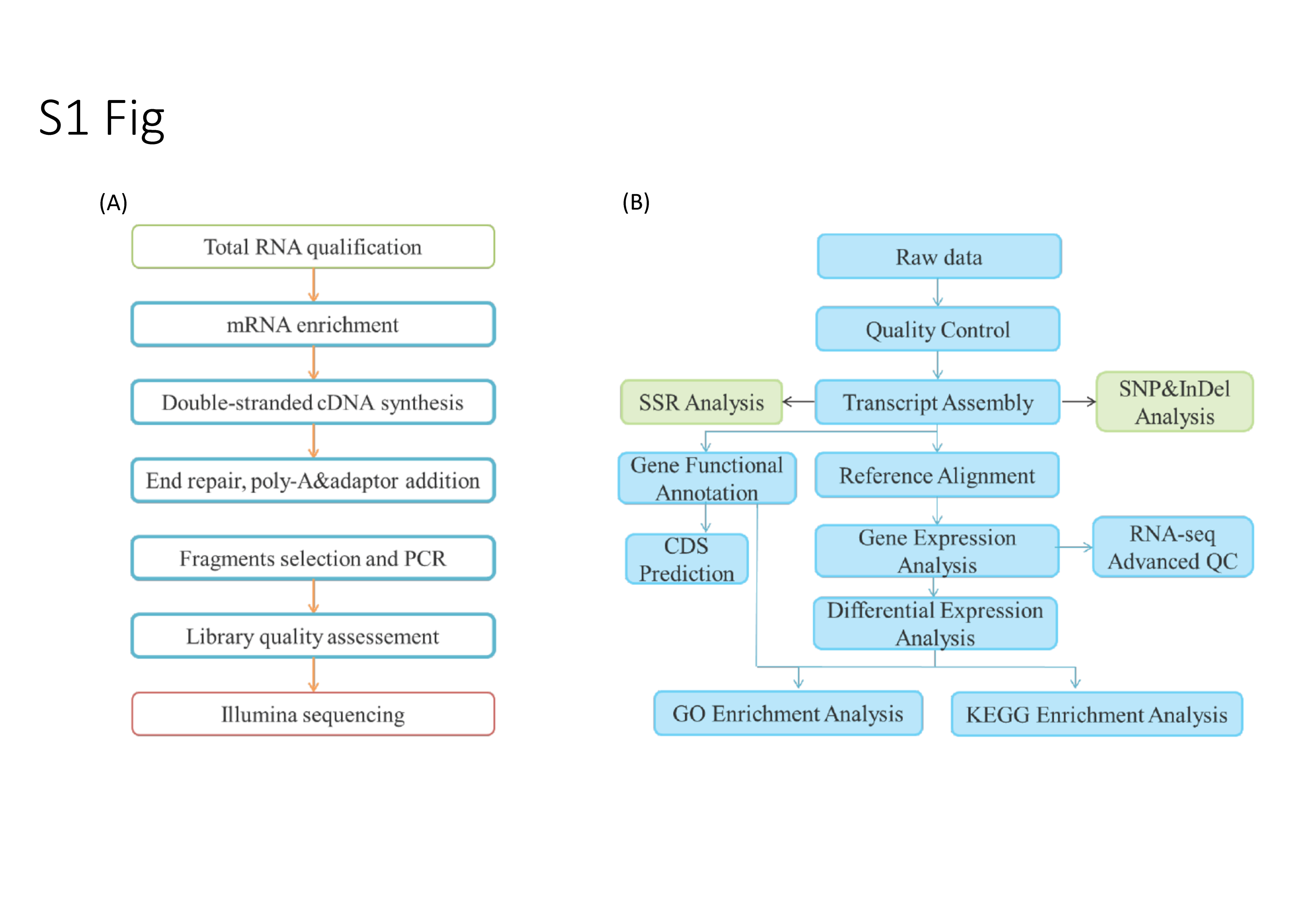

Supplement: S1 Fig — (TIF) [file pone.0247160.s001.tif]

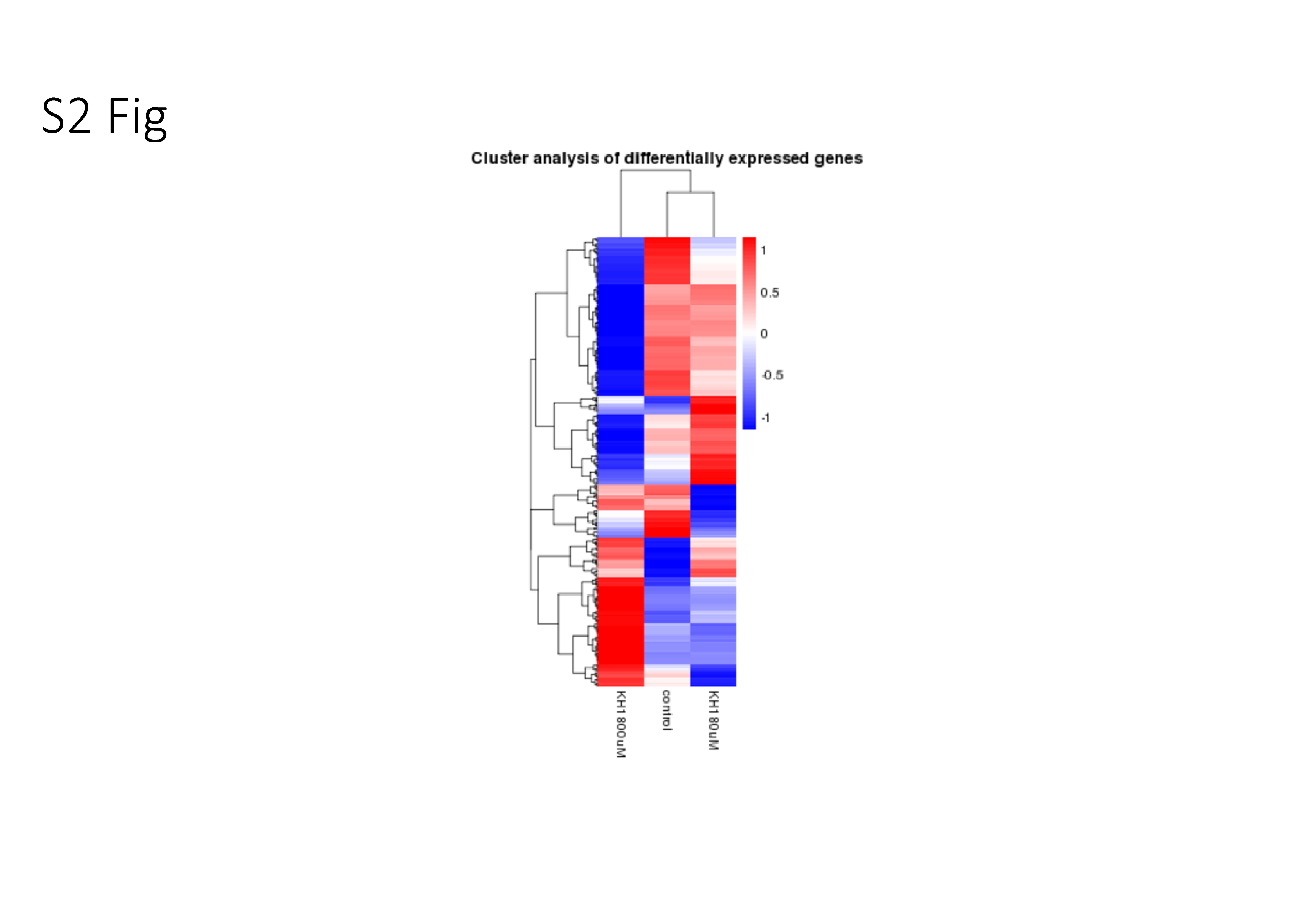

Supplement: S2 Fig — (TIF) [file pone.0247160.s002.tif]
