# Supplementary material for: Transcriptomic analysis elucidates the molecular processes associated with hydrogen peroxide-induced diapause termination in Artemia-encysted embryos
Source: PLoS One. 2021 Feb 19;16(2):e0247160. doi: 10.1371/journal.pone.0247160 (PMC7894940; doi:10.1371/journal.pone.0247160)
Supplement: S1 Table — (DOCX) [file pone.0247160.s003.docx]

S1 Table. Software and parameters used in this study.

| **Analysis** | **Software** | **Version** | **Parameter** | **Remark** |
| --- | --- | --- | --- | --- |
| Assembly | Trinity | r20140413p1 | min_kmer_cov:2, others are by default) | - |
| Hierarchical Clustering | Corset | v1.05 | -m 10 | remove redundancy |
| Gene Functional Annotation | Diamond | v0.8.22 | NR, Swiss-Prot: e-value = 1e-  5;KOG/COG: e-value = 1e-3 | NR, KOG/COG, Swiss-Prot |
|  | KAAS | r140224 | e-value = 1e-10 | KEGG Annotation |
|  | NCBI blast | v2.2.28+ | e-value = 1e-5 | NT Annotation |
|  | hmmscan | HMMER 3 | e-value = 0.01 | Pfam Annotation |
|  | blast2go | b2g4pipe_v2.5 | e-value = 1.0E-6 | GO Annotation |
| Mapping and Quantification | RSEM | v1.2.26 | bowtie2 mismatch 0 | mapping to Corset filtered transcriptome |
| Differential Expression Analysis | DEGSeq | 1.12.0 | padj&lt0.05 | For sample with bio-replicate using DESeq, samples without bio-replicate using DEGSeq.  EdgeR for specific conditions. |
|  | DESeq | 1.10.1 |  |  |
|  | edgeR | 3.0.8 |  |  |
| GO Enrichment | GOSeq, topGo | 1.10.0, 2.10.0 | Corrected P-Value&.lt0.05 | - |
| KEGG Enrichment | KOBAS | v2.0.12 | Corrected P-Value&.lt0.05 | - |
