# Supplementary material for: Transcriptomic analysis elucidates the molecular processes associated with hydrogen peroxide-induced diapause termination in Artemia-encysted embryos
Source: PLoS One. 2021 Feb 19;16(2):e0247160. doi: 10.1371/journal.pone.0247160 (PMC7894940; doi:10.1371/journal.pone.0247160)
Supplement: S2 Table — (DOCX) [file pone.0247160.s004.docx]

S2 Table. Overview of the alignment situation

| **Sample name** | **Total reads** | **Total mapped** |
| --- | --- | --- |
| Control-001 | 29476690 | 22236410 (75.44%) |
| Control-002 | 45582458 | 35221016 (77.27%) |
| Control-003 | 46494910 | 35956652 (77.33%) |
| 180μM-001 | 48301960 | 36930210 (76.46%) |
| 180μM-002 | 27789164 | 21463612 (77.24%) |
| 180μM-003 | 30614124 | 23439400 (76.56%) |
| 1800μM-001 | 25645468 | 19230310 (74.99%) |
| 1800μM-002 | 27800996 | 21465974 (77.21%) |
| 1800μM-003 | 30451370 | 22801106 (74.88%) |

(1) Sample name

(2) Clean reads number.

(3) Total number of reads that can be mapped to the reference genome.
