# Supplementary material for: Transcriptomic analysis elucidates the molecular processes associated with hydrogen peroxide-induced diapause termination in Artemia-encysted embryos
Source: PLoS One. 2021 Feb 19;16(2):e0247160. doi: 10.1371/journal.pone.0247160 (PMC7894940; doi:10.1371/journal.pone.0247160)
Supplement: S3 Table — (DOCX) [file pone.0247160.s005.docx]

S3 Table. The database for gene annotation and the annotated percentage.

| Metric | Number of Unigenes | Percentage (%) |
| --- | --- | --- |
| Annotated in NR | 37190 | 32.6 |
| Annotated in NT | 9114 | 7.99 |
| Annotated in KO | 19425 | 17.03 |
| Annotated in SwissProt | 31108 | 27.27 |
| Annotated in PFAM | 35927 | 31.49 |
| Annotated in GO | 36221 | 31.75 |
| Annotated in KOG | 21262 | 18.64 |
| Annotated in all Databases | 4748 | 4.16 |
| Annotated in at least one Database | 47020 | 41.22 |
| Total Unigenes | 114057 | 100 |
